# Betaine supplement alleviates hepatic triglyceride accumulation of apolipoprotein E deficient mice via reducing methylation of peroxisomal proliferator-activated receptor alpha promoter

**DOI:** 10.1186/1476-511X-12-34

**Published:** 2013-03-13

**Authors:** Lijun Wang, Li Chen, Yaozong Tan, Jun Wei, Ying Chang, Tianru Jin, Huilian Zhu

**Affiliations:** 1 Guangdong Provincial Key Laboratory of Food, Nutrition and Health, Sun Yat-Sen University, 74th Zhongshan Road II, Guangzhou, 510080, People’s Republic of China; 2 Department of Medicine and Physiology, University of Toronto, Toronto, Canada

**Keywords:** Betaine, *PPAR alpha*, Fatty liver, DNA methylation

## Abstract

**Background:**

Betaine is a methyl donor and has been considered as a lipotropic effect substance. But its mechanism remains unclear. Hepatic steatosis is associated with abnormal expression of genes involved in hepatic lipid metabolism. DNA methylation contributes to the disregulation of gene expression. Here we hypothesized that betaine supplement and subsequent DNA methylation modifications alter the expression of genes that are involved in hepatic lipid metabolism and hence alleviate hepatic triglyceride accumulation.

**Methods:**

Male wild-type (WT) C57BL/6 mice (*n* = 6) were fed with the AIN-93 G diet. *ApoE*^*−/−*^ mice (n = 12), weight-matched with the WT mice, were divided into two groups (*n* = 6 per group), and fed with the AIN-93 G diet and AIN-93 G supplemented with 2% betaine/100 g diet. Seven weeks after the intervention, mice were sacrificed. Liver betaine, choline, homocysteine concentration were measured by HPLC. Liver oxidants activity and triglyceride level were assessed by ultraviolet spectrophotometry. Finally, hepatic *PPAR alpha* gene and its target genes expression levels and the methylation status of the *PPAR alpha* gene were determined.

**Results:**

*ApoE*^*−/−*^ mice had higher hepatic triglyceride and lower GSH-Px activity when compared with the WT mice. Betaine intervention reversed triglyceride deposit, enhanced SOD and GSH-Px activity in the liver. Interestingly, mice fed on betaine-supplemented diet showed a dramatic increase of hepatic choline concentration and a decrease of betaine and homocysteine concentration relative to the WT mice and the *ApoE*^*−/−*^ mice absent with betaine intervention. Expression of *PPAR alpha* and *CPT1* were decreased and expression of *FAS* was markedly increased in *ApoE*^*−/−*^ mice. In parallel, *PPAR alpha* promoter methylation level were slightly increased in *ApoE*^*−/−*^ mice though without significance. Betaine supplement upregulated expression of *PPAR alpha* and its target genes (*CPT1*, *CYP2E1*) and reversed hypermethylation of *PPAR alpha* promoter of *ApoE*^*−/−*^ mice. Furthermore, *PPAR alpha* methylation was positively correlated with hepatic betaine concentration.

**Conclusions:**

Our findings indicate that betaine supplement could alleviate hepatic triglyceride accumulation and improve antioxidant capacity by decreasing *PPAR alpha* promoter methylation and upregulating *PPAR alpha* and its target genes mRNA expression.

## Background

Triglyceride (TG) accumulation in hepatocytes is considered the primary manifestation of fatty liver disease [1]. In addition, the increase of liver triglyceride deposit is associated with obesity, hepatocellular carcinoma [2] and atherosclerosis [3]. Therefore, relieving hepatic lipid accumulation is viewed as potentially promising strategy for the prevention of many chronic diseases. The pathogenesis of hepatic TG deposit is incompletely understood. A previous study by others has shown that upregulation of genes expression for de novo lipogenesis (*FAS*, fatty acid synthase; *ACC*, acetyl-CoA carboxylase) and downregulation of genes expression for fatty acid oxidation (*PPARα*, peroxisomal proliferator-activated receptor *alpha*; *CPT1*, carnitine palmitoyl transferase I; *UCP2*, uncoupling proteins; *ACOX*, acyl-CoA oxidase; *CYP2E1*, cyto-chrome P450 2E1) are involved in the onsets of triglyceride accumulation in the liver [4].

Betaine, a methyl donor, is a naturally occurring compound in common foods, such as wheat bran, wheat germ, spinach, pretzels, shrimp and wheat bread etc. [5]. In vivo, betaine can also be produced by oxidation of choline, and it serves as an effective methyl donor for remethylating homocysteine (Hcy) into methionine (Met). DNA methylation in the CpG islands is one of the epigenetic mechanisms to regulate gene expression. Choline and methionine deficiency in diet are closely associated with biological functions by differentially methylated and inversely expressed genes in the tissue [6-8]. Mice with hyperhomocysteinemia, induced by heterozygous cystathionine β-synthase deficiency, show diminished methylation capacity and hypermethylation silencing of *Fads2* mRNA expression which contribute to the impaired transport of TG [9]. High-fat diet can exacerbate methyl donors deficiency [10] and strikingly produce high level of serum Hcy, which may promote hypermethylation of *MTTP* gene and down-regulation of its expression, resulting in the hindrance to assembly lipoprotein and export lipid from liver [11]. It has become clear that *PPARα* can regulate the transcription of a suite of genes encoding enzymes in hepatic mitochondrial (*CPT1*, *UCP2*) and extramitochondrial (*ACOX*, *CYP2E*) fatty acid oxidation. Mice deficient in *PPARα* is demonstrated as a useful mouse model of fatty liver because of its important role in fatty acid oxidation and alleviation of hepatic TG [12]. Although An accumulating clinical and experimental evidences suggest that betaine is a lipotropic substance [13-15], the DNA methylation mechanism remains to be clearly defined.

In the present study, We attempt to investigate betaine supplement undergoing improvement on lipid metabolism and antioxidant capacity through changes in methylation level of *PPARα* promoter and expression of *PPARα* and its target genes(*CPT1*, *UCP2*, *ACOX*, *CYP2E*) in *ApoE*^*−/−*^ mice .

## Results

### Effect of betaine supplement on body weight and liver weight

**Figure 1:**
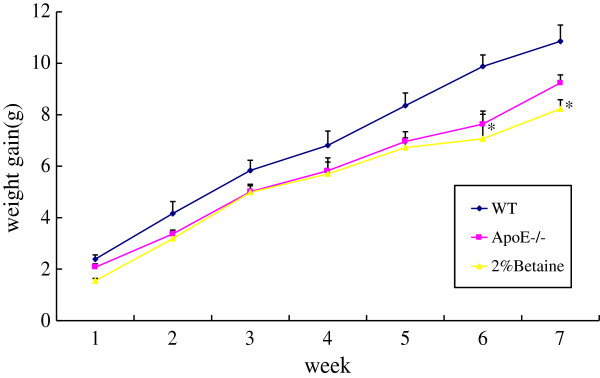
**Effects of betaine supplement on body weight gain.** WT mice were fed with the AIN-93 G diet, while *ApoE*^*−/− *^ mice were fed with the AIN-93 G in the absence and presence of 2% betaine. Values are means ± SE (n = 6). These data were tested by ANOVA. *P < 0.05 vs WT group.

Body weight was matched before grouping. Absolute body weight of each mouse from each group was measured weekly and summarized in Figure 1. As anticipated, body weight was increased after experiment initiation, gaining most rapidly in WT mice. No significant difference of body weight gain was found among groups, although the body weight gain in betaine-supplemented *ApoE*^*−/−*^ mice was significant lower than that in WT mice after 6 weeks. Additionally, there were no significant differences in liver weight (*F* = 2.422, *P* = 0.075) and liver/body weight ratio (*F* = 0.533, *P* = 0.712) among the mice fed with or without betaine at 7 weeks (data not shown). These results suggested that betaine supplement had no significant influence on either the liver weight or the body weight.

### Betaine supplement alleviates hepatic TG accumulation and improves antioxidant capacity

**Figure 2:**
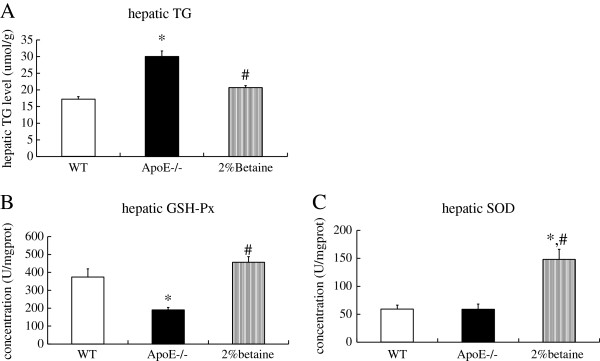
**Effects of betaine supplement on hepatic TG levels (A) and GSH-Px (B) and SOD (C) activity.** WT mice were fed with the AIN-93 G diet, while *ApoE*^*−/− *^ mice were fed with the AIN-93 G in the absence and presence of 2% betaine. Values are means ± SE, *n* = 6. *P < 0.05 vs WT group, # *P* < 0.05 vs *ApoE*^*−/−*^ group.

As anticipated, *ApoE*^*−/−*^ mice fed with the AIN-93 G diet showed significantly higher hepatic TG content than that in the WT mice. After supplemented with betaine, hepatic TG level was significantly reduced (*F* = 19.040, *P* < 0.001) (Figure 2A). Hepatic GSH-Px activity in the *ApoE*^*−/−*^ mice was significantly lower than that in the WT mice, while betaine supplement strikingly increased GSH-Px activity (*F* = 9.696, *P* < 0.001) (Figure 2B). Consistently, betaine feeding led to an significant elevation of hepatic SOD activity (*F* = 8.865, *P* < 0.001) (Figure 2C). We hence suggested that betaine intervention was able to ameliorate hepatic TG deposit and improve antioxidant capacity in the *ApoE*^*−/−*^ mice.

### Effect of betaine supplement on liver Betaine, Choline and Hcy concentration

**Figure 3:**
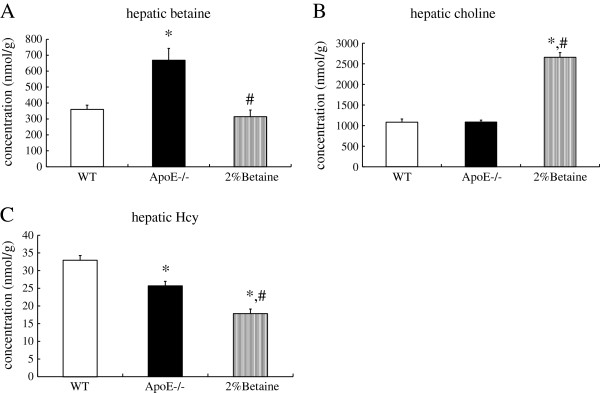
**Effects of betaine supplement on liver betaine (A), choline (B) and Hcy (C) levels.** WT mice were fed with the AIN-93 G diet, while *ApoE*^*−/− *^ mice were fed with the AIN-93 G in the absence and presence of 2% betaine. Values are means ± SE, *n* = 6. *P < 0.05 vs WT group, # *P* < 0.05 vs *ApoE*^*−/−*^ group.

Hepatic betaine concentrations in the *ApoE*^*−/−*^ mice were markedly higher and hepatic Hcy were significantly lower when compared with the WT mice. For the *ApoE*^*−/−*^ mice, betaine supplement normalized hepatic betaine levels (*F* = 11.014, *P* < 0.001) (Figure 3A), significantly increased hepatic choline levels (*F* = 36.492, *P* < 0.001) and reduced Hcy levels (*F* = 11.516, *P* < 0.001) (Figure 3B and 3C).

### The effect of betaine supplement on the expression of lipid metabolism related genes

**Figure 4:**
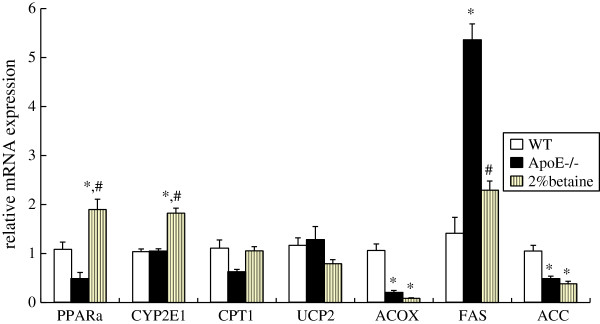
**Effect of betaine supplement on the expression of selected key genes involved in lipid metabolism.** WT mice were fed with the AIN-93 G diet, while *ApoE*^*−/− *^ mice were fed with the AIN-93 G in the absence and presence of 2% betaine. Values are means ± SE (n = 6). *P < 0.05 vs WT group, # *P* < 0.05 vs *ApoE*^*−/−*^ group.

In the *ApoE*^*−/−*^ mice, hepatic *PPARα* and *CPT1* expression levels showed a trend of reduction when compared with WT mice, although the difference did not reach the statistical difference level. Significant up-regulation of *PPARα* (3.93 folds) and C*YP2E1* (1.82 folds), however, was detected in the *ApoE*^*−/−*^ mice by betaine supplementation (*F* = 12.494, *P* < 0.001) (Figure 4). Meanwhile, hepatic *FAS* mRNA levels in the *ApoE*^*−/−*^ mice were significantly higher than the WT controls, while betaine treatment significantly attenuated its expression levels. Despite these changes, betaine supplement did not exert effect on expression of other lipogenic and oxidative genes, such as *ACC, UCP2, ACOX*. These data suggested that betaine supplement selectively altered expression of certain genes. These results prompted us to further examine whether gene expression change in response to betaine supplement was influenced by DNA methylation.

### B**etaine supplement decreases methylation level of hepatic*PPARa*promoter**

**Figure 5:**
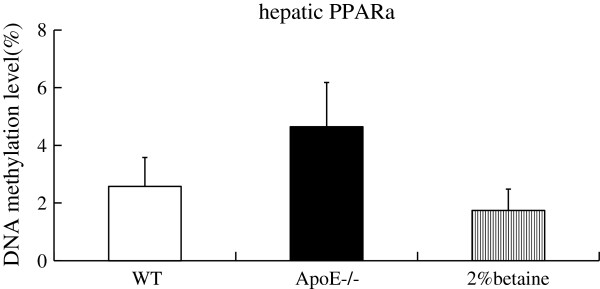
**Detection of aberrant promoter methylation of the *PPARa *gene by real-time quantitative MSP.** WT mice were fed with the AIN-93 G diet, while *ApoE*^*−/− *^ mice were fed with the AIN-93 G in the absence and presence of 2% betaine.

**Figure 6:**
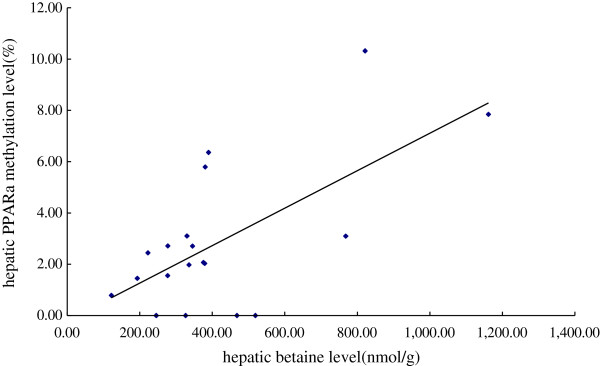
**Linear correlation analysis of DNA methylation level of *PPARα *promoter vs the hepatic betaine levels.** WT mice were fed with the AIN-93 G diet, while *ApoE*^*−/− *^ mice were fed with the AIN-93 G in the absence and presence of 2% betaine.

We then chose the *PPARα* as target gene and utilized a real-time quantitative MSP method to quantitatively assess the methylation levels of *PPARα* promoter region in the liver. The average methylation in the *PPARα* gene promoter region of the *ApoE*^*−/−*^ mice was 4.64%,compared with 2.58% in the WT mice.Betaine supplementation showed a trend of decreasing the methylation level, although the difference didn’t reach a statistical difference level (Figure 5). Reduced methylation levels of *PPARα* promoter was accompanied by increased expression of the *PPARα* gene. A positive relationship was also found between the hepatic betaine levels and the methylation levels of the *PPARα* promoter (*β* = 0.572, *P* = 0.005) (Figure 6). These results collectively suggested that the DNA methylation at the *PPARα* gene promoter could be regulated by betaine supplementation.

## Discussion

The current study was designed to determine whether betaine intervention improves hepatic TG metabolism and antioxidant capacity in the *ApoE*^*−/−*^ mice. There are two novel findings of this study. The first one is that betaine supplement can reduce hepatic TG content and improve antioxidant capacity,associated with the normalization of the expression of *PPARα, CYP2E1,CPT1* and *FAS*. Secondly,we show here a tendency of increased DNA methylation in the *PPARα* promoter in the *ApoE*^*−/−*^ mouse liver while betaine supplement is able to reverse the hypermethylation of the *PPARα* promoter.

*ApoE*^*−/−*^ mouse is the classical modle of AS and dyslipidemia, characterized by high levels of plasma cholesterol and/or TG or low level of HDL, in addition, this mouse model indicates some incline to express fatty liver [16]. Our previous study [17] has shown that betaine has an anti-atherosclerosis effect in the *ApoE*^*−/−*^ mice. We further evidenced that betaine intervention effectively relieved hepatic triglyceride deposit in the present study. Hepatic steatosis generated by environmental and genetic factors have been proved to influence genes expression involved in the process of lipid metabolism [4,8,9,11,18]. Fatty acids in the liver are derived from the de novo lipogenesis and the uptake from plasma, and catabolized by oxidation or exportation in the form of VLDL-TG. Fatty acid oxidation occurs mainly in the mitochondria, in which ATP is generated simultaneously. When the impairment of antioxidant capacity in mitochondria generated by excessive fatty acid occurs, the alternative peroxisomal β-oxidation and microsomal ω-oxidation will be activated which inevitably lead to the production of reactive oxygen species (ROS), including hydrogen peroxide (H_2_O_2_) and superoxide. *PPARα* is highly expressed in the liver and it is well-known to promote fatty catabolism by up-regulation of genes in mitochondral and extramitochondral fatty acid oxidation. The expression of hepatic *PPARα* expression has shown to be down-regulated in NAFLD patients [4], *ApoE*^*−/−*^ mice also exhibited reduced hepatic *PPARα* expression compared with WT mice though without statistical significance in our present study. As we have expected, betaine supplement in *ApoE*^*−/−*^ mice dramatically upregulated *PPARα* gene expression. The expression of *CPT1*, a key rate-limiting enzyme in mitochondral oxidation, just mirrored the alteration of *PPARα* expression in response to betaine treatment. *CYP2E1* is one of the targets of *PPARα,* a key enzyme in the microsomal ω-oxidation. *CYP2E1* overexpression in the liver is a definite factor for oxidative stress [19]. In the current study, we found that betaine treatment increased *CYP2E1* expression which was paralleled by increased *PPARα* expression. We hence further investigated the activity of SOD and GSH-Px since these two enzymes are involved in the elimination of ROS derived from fatty acid oxidation. We observed a diminished antioxidant capacity in *ApoE*^*−/−*^ mice, but elevated antioxidant activity in the liver of the *ApoE*^*−/−*^ mice fed with betaine. These results indicated that *CYP2E1* overexpression is unable to induce oxidative stress, but how *ApoE*^*−/−*^ mice showed elevated hepatic TG and diminished antioxidant capacity and how betaine supplement reversed it? *FAS* and *ACC* are two key enzymes in the process of fatty acid synthesis. The current results revealed that expression of *FAS* was increased by five folds in the liver of the *ApoE*^*−/−*^ mice when compared with WT controls, while betaine treatment strikingly repressed it. Accumulation of excessive hepatic TG and consequent oxidative stress have been confirmed to be the two common “hits” in the development of NAFLD [1]. Betaine treatment significantly increased *PPARα, CPT1* and *CYP2E1* expression and reduced the expression of *FAS*, the latter effect impaires fatty acid synthesis so as not to supply substrate for lipid peroxidation, and enhanced mitochondrial oxidation is sufficiently powerful to prevent TG deposit and oxidative stress.

The precise mechanisms by which betaine up regulates gene expression remain largely unclear. DNA methylation is one of the epigenetic modifications involved in the regulation of gene expression and hence versatile biological functions. Significant loss of global methylation was observed in the *ApoE*^*−/−*^ mice by other investigators [20]. It is widely accepted that cells could undergo global DNA hypomethylation but simultaneously possess hypermethylation of a specific gene promoter, while the expression of a gene is usually silenced or attenuated when its promoter is heavily methylated. Methyl-deficient diet has been evidenced to exhibit hypermethylation promoter of genes involved in lipid metabolism which has inverse relationship with gene expression [8]. Betaine with its three chemically reactive methyl groups could serve as a methyl donor for DNA, RNA, histone and proteins, and its supplement may reverse alterations in DNA methylation induced by methyl deficiency. Here we investigated for the first time whether *PPARα* promoter methylation and corresponding gene expression can be affected by betaine supplement. The results of quantitative MSP assay showed that the methylation level of *PPARα* promoter was higher in the *ApoE*^*−/−*^ mice when compared with that of WT mice, while betaine treatment showed a tendency to attenuate it. In attempt to explore the underlying mechanism, we did further analysis and found out that *PPARα* promoter methylation level was positively correlated with hepatic betaine concentration.

In present study, betaine supplement significantly decreased Hcy concentration and increased hepatic choline concentration. Unexpectedly, betaine treatment decreased hepatic betaine concentration as well. Endogenous betaine is oxidized from choline by two steps. Choline dehydrogenase (CHDH) mediates the first committed step for the formation of betaine aldehyde from choline, which is further catalyzed into betaine by betaine aldehyde dehydrogenase (BADH). Once betaine is formed, it serves as methyl donor for remethylating Hcy into Met by betaine-homocysteine methyltransferase (BHMT). Hepatic CHDH is rarely affected by dietary methionine and choline [21], but it has been shown to be competitively inhibited by both betaine aldehyde and betaine [22]. BHMT is expressed primarily in the liver of humans and mice [23]. Betaine-alone supplementation results in a 2-fold elevation of hepatic BHMT levels and activities [24]. Thus, betaine-elicited repression of CHDH as well as of activation BHMT can collectively increase the catabolism but decrease the synthesis of betaine in the liver. Our finding that betaine treatment dramaticly increased choline level and reduced Hcy level in the liver also confirmed this effect. This is among potential explainations why liver betaine levels in the betaine-supplemented *ApoE*^*−/−*^ mice dropped to the levels that were comparable with the levels in the WT mice. We hence suggest that hepatic betaine depletion plays a role in the hypomethylation of *PPARα* promoter.

On the other hand, choline, another methyl donor, was observed to dramaticly increase by betaine intake. Betaine has been proved to have choline-sparing effect [25]. Choline is a basic component for the synthesis of Phosphatidylcholine (PC) which takes part in the assembly of mature VLDL. Betaine-containing diet could enhance the synthesis of hepatic SAM as well as the activity of phosphatidylethanolamine methyltransferase (PEMT), which subsequently facilitates the metabolic conversion of phosphatidylethanolamine (PE) to form PC [24]. *ApoE*^*−/−*^ mice is characterized by lipid accumulation and the development of hepatic steatosis. As a result, more choline are available to increase PC synthesis and PC:PE ratio in VLDL, thereby promoting VLDL secretion and lipid transport [26]. These results suggest that betaine supplement exert important effects on the pool of related metabolites in the liver,whereby reduced betaine concentration in the liver connot supply enough substrate for DNA methylated modification and may selectively decrease the methylation statue of certain gene involved in lipid metabolism.

## Conclusion

Taken together, the present study reports for the first time that betaine intervention reduces hepatic TG content and improves antioxidant capacity in the *ApoE*^*−/−*^ mouse model. This is partially achieved by upregulating *PPARα* gene expression, as a result of hypomethylation of its promoter. These findings provided a novel insight into the important lipotropic effect of betaine. Due to its fundamental important role as a methyl-donor, betaine is gradually become a new target for the intervention of many chronic metabolic diseases. The hepatic and vascular protective effects of betaine we found provide promising insight into the human nutrition. Our present study has also build up a strong basis for further evaluation of potential mechanisms of betaine in epigenetic studies.

## Methods

### Chemicals

Choline chloride, Betaine glycine, d9-Betaine (internal standard), d9-choline (internal standard), trichloroacetic acid (TCA), tris (2-carboxylethyl) phosphine (TCEP), ammonium-7- fluorobenzo-2-oxa-1, 3-diazole-4-sulfonic acid (SBD-F), mercaptopropionylglycine (MPG), mineral oil were purchased from Sigma Chemicals. Methanol, chloroform, acetonitrile, formic acid were HPLC grade and obtained from Merck Chemical.

### Animals and diet

Male wild-type (WT) C57BL/6 mice (*n* = 6) and *ApoE*^*−/−*^ mice on the C57BL/6 background (*n* = 12) were obtained from the Jackson Laboratories (Bar Harbor, ME, USA). All mice were acclimated on a standard AIN-93 G diet for one week. The *ApoE*^*−/−*^ mice were weight-matched and divided into two groups (*n* = 6 per group) feeding with the AIN-93 G diets supplemented with 0, 2 g betaine/100 g diet and the WT mice continue to consume the AIN-93 G diet. All mice were housed at a constant temperature, humidity and 12 hR light/dark cycle, and freely accessed to food and water for 7 weeks. Body weight and consumption of diet were recorded weekly. Mice were anesthetized and sacrificed after the experiment, liver were excised and stored at −80°C. This study was approved by the Institutional Animal Care and Use Committee at Sun Yat-Sen University.

### Determination of betaine and choline concentration in the animal tissue

One hundred mg frozen liver tissue were homogenized in 600 μl methanol/chloroform (2:1,V/V) using an Ultrasonic Cell Disruptor (PRO scientific Inc. USA). The mixture was vortex-mixed vigorously and left at −20°C overnight. After the extraction, the mixture was centrifuged at 1500 g for 5 min. The supernatants were drawn and transferred to new tubes. The residuals were mixed with 300 μl methanol/chloroform/water (2:1:0.8,V/V/V) and centrifuged at 1500 g for 5 min. The supernatants from the two extractions were pooled. One hundred μl chloroform and 100 μl water were added into the mixture to form two phases. After a centrifugation, the aqueous phase was taken by a syringe, dried by N_2_ Blowing Concentrator (Organomation Associates Inc, America) and dissolved in 20 μl water. The dissolved water were treated with 800 μl methanol, 100 μl acetonitrile containing 10 μM internal standard and centrifuged at 6000 g for 2 min to precipitate protein. Finally the supernatants were injected into high performance liquid chromatography-tandem mass spectrometry (HPLC-MS) to analysis [27].

### Determination of Hcy concentration in the animal tissue

Twenty five mg frozen liver tissue was homogenized in 200 μl cold PBS using an Ultrasonic Cell Disruptor (PRO scientific Inc, USA). The homogenized tissue suspension was treated with 50 μl TCEP (120 mg/ml) and 60 μl MPG (10 μM), vortex-mixed thoroughly, and incubated at 37°C for 30 min. After adding 125 μl TCA (100 g/L with 1 mM EDTA) and vortex-mixing thoroughly, the precipitated proteins were immediately centrifugated at 13000 g for 10 min. One hundred μl supernatant was incubated with 125 μl borate buffer (0.125 M, pH 9.5, with 4 mM EDTA), 10 μl NaOH solution (1.55 M) and 50 μl SBD-F solution (1 g/L in borate buffer) at 60°C for 60 min, placed in the room temperature to cool down, and then samples (10 μl) were injected into HPLC system to analysis [28].

### Bisulfite conversion of DNA

Genomic DNA was isolated from the liver tissue with the TIANamp Genomic DNA Kit (TIANGEN, Beijing, China). Treatment of DNA with bisulfite would result in the conversion of unmethylated cytosines to uracils, while methylated cytosines would remain unaltered. Briefly, 5.5 μL of 3 M NaOH was added into 1 ~ 2 μg of DNA (50 μL volume) and incubated at 42°C for 20 min. Following the incubation step, 520 μL of 3.6 M NaHSO3 (pH 5.0) and 30 μL 10 mM hydroquinone were added to each sample (fresh and avoiding exposure to light). All reagents were softly mixed, centrifuged and covered by mineral oil, then incubated at 53°C for 16 h. The bisulfite-converted DNA was purified by Wizard DNA Clean-Up System (Promega, USA). Finally DNA was eluated with 54 μL DDW at 80°C. 6 μL of 3 M NaOH was added into each sample and incubated at 42°C for 15 min. Following the incubation, DNA was precipitated using 6 μl of 3 M sodium acetate and 30 μl 100% cold ethanol at −80°C for 3 h. Each sample was centrifuged 30 min at 12,000 g and the supernatant liquid was poured out. The bisulfite-converted DNA was washed by 70% ethanol, dried and finally resuspended in a total volume of 10 μl.

### Real-time quantitative MSP

The chemically modified DNA was subsequently used as a template for a QMSP method to determine the methylation level of the selected CpG dinucleotides in the *PPAR*α promoter. For the methylation-specific PCR experiment, two pairs of primers were designed to amplify either methylated or unmethylated sequences: one pair was specific for bisulfite-modified methylated DNA (M primers) and the other pair was specific for bisulfite-modified unmethylated DNA (U primers). The sequence was retrieved from NCBI at http://www.ncbi.nlm.nih.gov/gene? with the **accession number** [**Genbank:NM_001113418.1]**. Primers were designed via methprimer on line (http://www.urogene.org/methprimer/) and the sequences were shown in Table 1. NIH 3 T3 mouse genomic DNA and fully methylated NIH 3 T3 mouse genomic DNA (New England Biolabs, USA) were separately used as fully unmethylated and methylated positive control. Negative water blanks were also included. For each 20 μl Real-time quantitative PCR, 10 μl 2 × SYBR® Premix Ex Taq TMII, 0.2 μl of 10 μM forward primer, 0.2 μl of 10 μM reverse primer, 0.4 μl of 50 × ROX, 2 μl DNA template and 7.2 μl water were used. All of the regents could be obtained from SYBR® Premix Ex Taq TM II Kit(Takala). For the *PPARa* M and *PPARa* U Real-time quantitative MSP, the condition was 94°C for 3 min followed by 40 cycles of 94°C for 30 s, 55°C for 31 s, 72°C for 30 s. Data acquisition at annealing phase were analysed. After amplification, a melt curve was generated to confirm the specificity of PCR product. Serial dilutions of fully unmethylated amplified PCR product and fully methylated amplified PCR product were used to establish calibration curve. Data were collected by the Applied Biosystems 7500 fast real-time PCR system and analysed using 7500 software V2.0.1. The methylation index in a sample and the completeness of bisulfite conversion was estimated as previously described by Y. M. Dennis Lo [29]. Each sample was tested in duplicate. The melt curves in the present study confirmed stable and specific PCR products in both methylated (M) and unmethylated (U) systems. The linearity correlation coefficient of the standard curve of M system was 0.999, which demonstrated a large dynamic range and accuracy of real-time quantitative PCR. Similar results were obtained from U system as well.

**Table 1:** Oligo-nucleotide primer pairs for detecting of genes promoter methylation and mRNA expression

**Gene symbol**	**Primer sequence(5**^**′**^**-3**^**′**^**)**	**Anneal temperature**	**Amplicon size**
*PPARa* M	F: TTTTTA**CG**AATTTT**CG**GGATT**C**	55	164
	R: AAAAAATA**CG**CCTTAA**CGCG**		
*PPARa* U	F: TTTTTTTATGAATTTTTGGGATTTG	55	169
	R: TAAAAAAAATACACCTTAACACACA		
*PPARa*	F: GGGCAAGAGAATCCACGAAG	60	91
	R: GTTGTTGCTGGTCTTTCCCG		
*CYP2E1*	F: TGCAGTCCGAGACAGGATGA	60	82
	R: GGAAGGGACGAGGTTGATGA		
*CPT1*	F: AGCACACCAGGCAGTAGCTT	60	144
	R: AGGATGCCATTCTTGATTCG		
*UCP2*	F: TCTCCTGAAAGCCAACCTCAT	60	272
	R: GCTGCTCATAGGTGACAAACAT		
*ACOX*	F: AGGTTCAGTCGGGGAAGCTGG	60	433
	R: ATCTGAGCCCCTGTGATGATG		
*FAS*	F: GCTGCGGAAACTTCAGGAAAT	60	84
	R: AGAGACGTGTCACTCCTGGACTT		
*ACC*	F: CACTGTGAACATGTGGAGG	60	155
	R: AGGCTGATGGTGATGACC		
*β-Actin*	F: GGGTCAGAAGGACTCCTATG	60	90
	R: GTAACAATGCCATGTTCAAT		

Sequences with bold font mean CpG site in the primers.

### RNA isolation and RT-PCR

Total RNA was isolated from the liver tissue with the TRIZOL reagent (Invitrogen, USA) according to the manufacturer’s instruction. The concentration and quality of total RNA was assessed by spectrophotometer, and its integrity was tested on agarose gels. Two μg RNA was reversely transcripted into cDNA with a commercial kit (Invitrogen, USA). Quantitative analysis of gene expression was performed by real-time polymerase chain reaction with an Applied Biosystems 7500 fast real-time PCR system. PCR primer information is summarized in Table 1.

### Hepatic biochemical analysis

Total hepatic lipid was extracted by the method presented by Folch and colleagues [30]. Hepatic TG concentrations were determined using commercially available kits (Biosino Bio-Technology and Science Inc, Beijing, China) on a automatic biochemistry analyzer (A25Biosystem, Spain). Hepatic Superoxide dismutase (SOD), glutathione peroxidase (GSH-Px) activities were determined using correspondent kits (Nanjing Jiancheng Bio Inc, Nanjing, China) with ultraviolet spectrophotometry.

### Statistic analysis

All results were presented as mean ± standard error (SE) and tested by the analysis of one way ANOVA followed by Bonferroni’s multiple comparison. Linear correlation was conducted to assess the relationship between hepatic betaine concentration and methylation level of *PPARα* gene. A two-tailed *P* < 0.05 was considered statistically significant.

## Abbreviations

ApoE−/−: *ApoE* deficient; WT: Wide type; TG: Triglyceride; GSH-Px: Glutathione peroxidase; SOD: Superoxide dismutase; Hcy: Homocysteine; PPARα: Peroxisomal proliferator - activated receptor alpha; CPT1: Carnitine palmitoyl transferase I; UCP2: Uncoupling proteins; ACOX: Acyl-CoA oxidase; CYP2E1: Cyto-chrome P450 2E1; FAS: Fatty acid synthase; ACC: Acetyl-CoA carboxylase

## Competing interests

The authors have no conflict of financial interests to declare.

## Authors’ contributions

LW participated in the whole process of the study, including carrying out molecular genetic experiments, data analyses and drafting manuscript. LC participated in the animal feeding experiment. YT and YC were in charge of determining hepatic metabolites by HPLC system. JW was in charge of assessing the levels of hepatic lipids and oxidants activities. TJ participated in the manuscript revising and language modifications. HZ was responsible for designing the research and helped to draft and revise manuscript. All authors read and approved the final manuscript.

## Source of funding

This study was supported by grants from National Natural Science Foundation of China (81072302 and 81273050) and Natural Science Foundation of Guangdong Province, People’s Republic of China (10151008901000207).
